# Abstract images and words can convey the same meaning

**DOI:** 10.1038/s41598-018-25441-5

**Published:** 2018-05-08

**Authors:** Jan Rouke Kuipers, Manon Wyn Jones, Guillaume Thierry

**Affiliations:** 1 University of Stirling, Stirling, FK9 4LA United Kingdom; 2 Bangor University, Gwynedd, LL57 2AS United Kingdom

## Abstract

Intuitively, deriving meaning from an abstract image is a uniquely human, idiosyncratic experience. Here we show that, despite having no universally recognised lexical association, abstract images spontaneously elicit specific concepts conveyed by words, with a consistency akin to that of concrete images. We presented a group of naïve participants with abstract picture–word pairs construed as ‘related’ or ‘unrelated’ according to a preliminary norming procedure conducted with different participants. Surprisingly, the naïve participants with no prior exposure to the abstract images or any hints regarding their possible meaning, displayed a reaction time priming effect for ‘related’ *versus* ‘unrelated’ picture-word pairs. Critically, this behavioural priming effect, and an associated decrease in N400 mean amplitude indexing semantic priming, both correlated significantly with the degree of relatedness established in the preliminary norming procedure. Given that ratings and electrophysiological measures were obtained in different groups of individuals, our results show that abstract images evoke consistent meaning across observers, as has been shown in the case of music.

## Introduction

Abstract images, such as a works of art, are a powerful means of eliciting individualized emotional reactions and general impressions in the observer^1–3^. Yet, it is currently unknown how abstract images activate brain processes associated with *meaning*. Here, we used a simple picture–word semantic priming paradigm to examine whether abstract images are systematically linked to specific abstract concepts.

Semantic priming typically refers to the automatic processing advantage observed for words that are preceded by semantically related content^4,5^. In event-related potentials (ERPs), semantic priming is reflected by a modulation of N400 mean amplitude. The N400 is a negative deflection peaking between 350–500 ms post stimulus onset with a frontal-central to central-parietal scalp distribution, with unrelated stimulus pairs eliciting more negative N400 amplitudes than related ones^6,7^. A substantial literature documents ‘N400 effects’ in online linguistic analysis, but the effect is also robust to modality changes between context and target word^8^. Thus, picture-word priming experiments reliably show a processing advantage for target concrete words (e.g., *dog*) when the preceding context is congruent with the target word (e.g., a picture of a dog)^9,10^, consistent with the widely accepted theory that concrete concepts are represented by a rich network of visual-verbal representations^11^.

Whereas concrete images provide a visual referent for concrete concepts, analogous representations underpinning abstract images are less well understood. We do know that when faced with an initially uninterpretable image, the visual system is driven to bind correlated features in an attempt to create the illusion of unitary objects^12–14^. Indeed, abstract images depicted in artworks, for example, often comprise ‘supernormal’ stimuli –a composite caricature of the viewer’s previous visual experiences– to excite form areas in the brain more strongly than natural stimuli^15^. Even abstract images can therefore represent concrete concepts, but to our knowledge no previous study has examined whether abstract images also spontaneously elicit specific *abstract concepts*.

**Figure 1:**
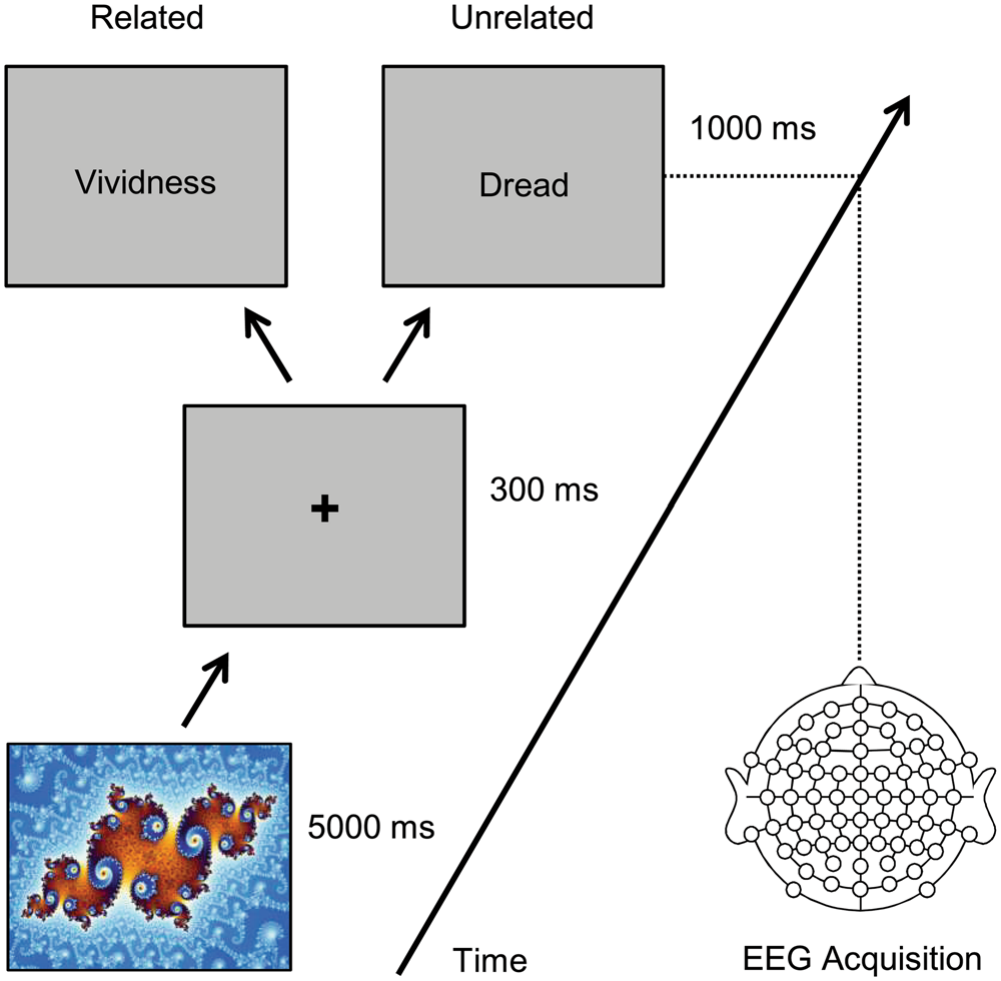
Trial procedure. Abstract images were presented full screen on a 17′′ monitor for 5 s. Following a brief interval with a central fixation cross (of 300 ms), a word was presented centrally for a duration of 1 s. Participants were required to make a semantic relatedness judgement while their ERP and behavioral responses were recorded. Displayed as the stimulus is Mandel, zoom 14, satellite julia island by Wolfgang Beyer (https://commons.wikimedia.org/wiki/File:Mandel_zoom_14_satellite_julia_island.jpg; Wikimedia Commons, CC-BY-SA 3.0; https://creativecommons.org/licenses/by/4.0/).

Here, we tested whether abstract images semantically prime abstract concepts. We measured participants’ N400 responses to target words preceded by an abstract picture, in which picture-word pairs had been previously categorized as ‘related’ or ‘unrelated’ on the basis of ratings provided by a different group of participants (Fig. 1). Whilst the semantic representations of abstract concepts are traditionally conceptualized as linguistic^16,17^, recent theorizing emphasizes the rich, multi-contextual array of sensory as well as linguistic episodic traces that give rise to concepts such as *dread*, *beauty*, *fury*, and *enlightenment*^18–22^. Assuming that undefinable forms presented in abstract images can also partially represent the sensory-visual experiences of abstract concepts, we hypothesized that ‘related’ picture-word pairs would elicit shorter RTs and reduced N400 amplitudes compared with ‘unrelated’ pictures. Alternatively, if regularities in ratings are underpinned by subjective, re-evaluative processing, we expected that any systematic trend in the initial rating study would not carry over to online measures of semantic processing collected in a different group of participants.

## Results

We first assessed the extent to which abstract images are systematically linked to abstract words. To this end, we collected abstract images from online databases and paired them with abstract words according to the experimenters’ consensus. These picture-word pairs were then presented online to a group of psychology students (*n* = 26) asked to rate picture–word relatedness on scale of 0–10. The highest scoring picture-word pairs (52 out of 100; *M rating*: 6.8; *SE*: 0.07, *range*: 6–8.3) were randomly re-associated and checked so as to create unrelated picture-word pairs. The two sets of picture-word pairs (related, unrelated) were subsequently rated as significantly different (t_(51)_ = 17.3, *p* < 0.001) by another group of participants (*n* = 48; *M related items rating*: 7.3; *SE* = 0.1; *range*: 4.8–8.6; *M unrelated items rating*: 3.4; *SE* = 0.1; *range*: 1.3–5.2), showing that abstract pictures and words were rated consistently as to their relatedness across different groups of participants.

**Figure 2:**
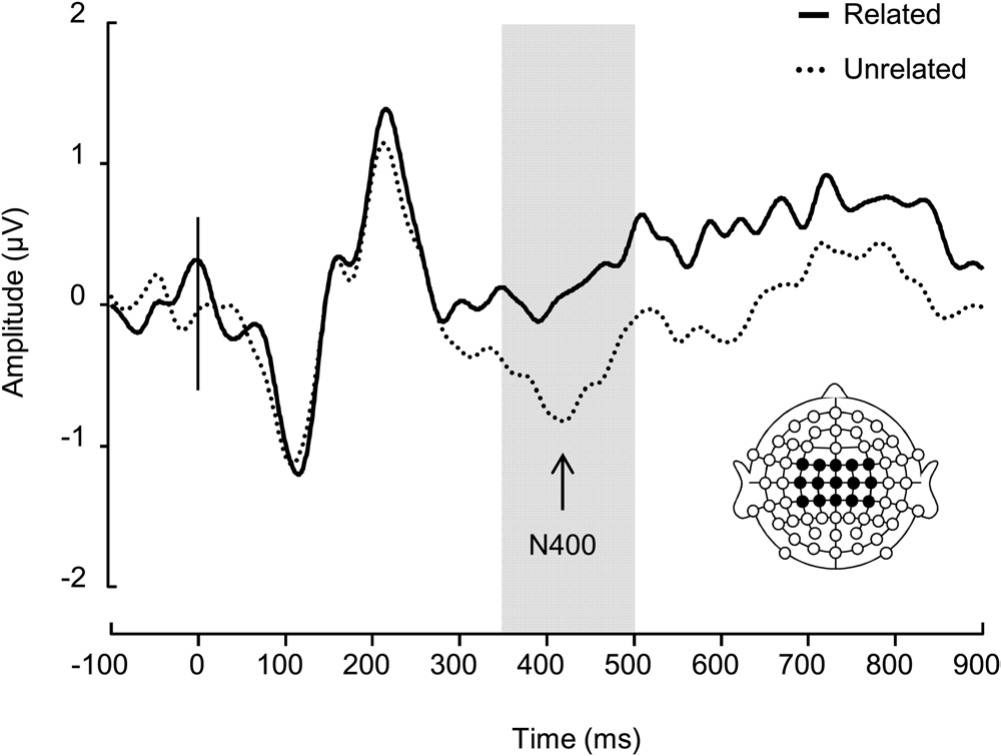
Grand average ERP waveforms. The average of 9 frontocentral, central, centroparietal electrodes (FC1-4 & Z, C1-4 & Z, & CP1-4 & Z). Word onset is indicated with the vertical line at 0 ms. The grey shaded area indicates the N400 with a more negative amplitude elicited by unrelated than related words.

Next, we tested whether the abstract images spontaneously elicited the meaning of its ‘related’ word in a semantic priming paradigm. Images were presented in isolation to a new group of participants (*n* = 20) whilst behavioral and brain responses to the related and unrelated word were collected (Fig. 1). On each trial participants indicated picture-word relatedness (related or unrelated) by executing a binary-choice button press. RTs were significantly longer (F_(1,17)_ = 6.4, *p* < 0.05, η = 0.27) and N400 amplitude (350–500 ms post word onset) was significantly more negative (F_(1,17)_ = 10.6, *p* < 0.01, η = 0.38) for ‘unrelated’ words as compared ‘related’ words (Fig. 2). Surprisingly, we found that RTs and mean N400 amplitude were significantly correlated (R = −0.49, *p* < 0.05). Given that RTs reflect all processes that lead to a response, including stimulus identification and response production, they rarely correlate with N400 amplitude which indexes semantic processing more directly^23^. Therefore, our results show that (a) the participants’ overt decision was largely based on semantic analysis, (b) longer responses were due to a semantic mismatch between the picture and the word, and (c) 5 seconds was ample viewing time for abstract images to spontaneously and consistently elicit the meaning of related words.

**Figure 3:**
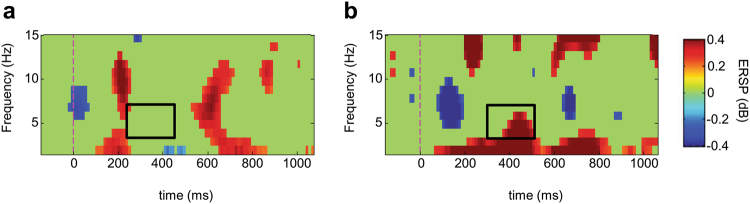
Log transformed event-related spectral perturbations of the related (left) and unrelated (right) words. Only significant changes (p < 0.05) from the baseline period (−200 to 0 ms relative to word onset) are colored other than green. Theta activity (3–7 Hz) was significantly increased in the unrelated compared to the related condition in the N400 time range (boxed areas; t_(1,84)_ = 3.1, *p* = 0.002).

Our interpretation of these observations was further supported by a time-frequency decomposition of single trial EEG data from which the phase-locked ERP data was removed^24^. This so called “induced” neural activity reflects neural oscillations that are mostly filtered out in the calculation of ERPs. Semantic priming^25^ in addition to error detection^26^, and working memory updating tasks^27^ typically result in an increase in theta activity over frontocentral electrode sites and, indeed, oscillations at electrode FCz showed a significant increase in theta activity for unrelated as compared to related words in the N400 time range (t_(1,84)_ = 3.1, *p* < 0.01; Fig. 3).

**Figure 4:**
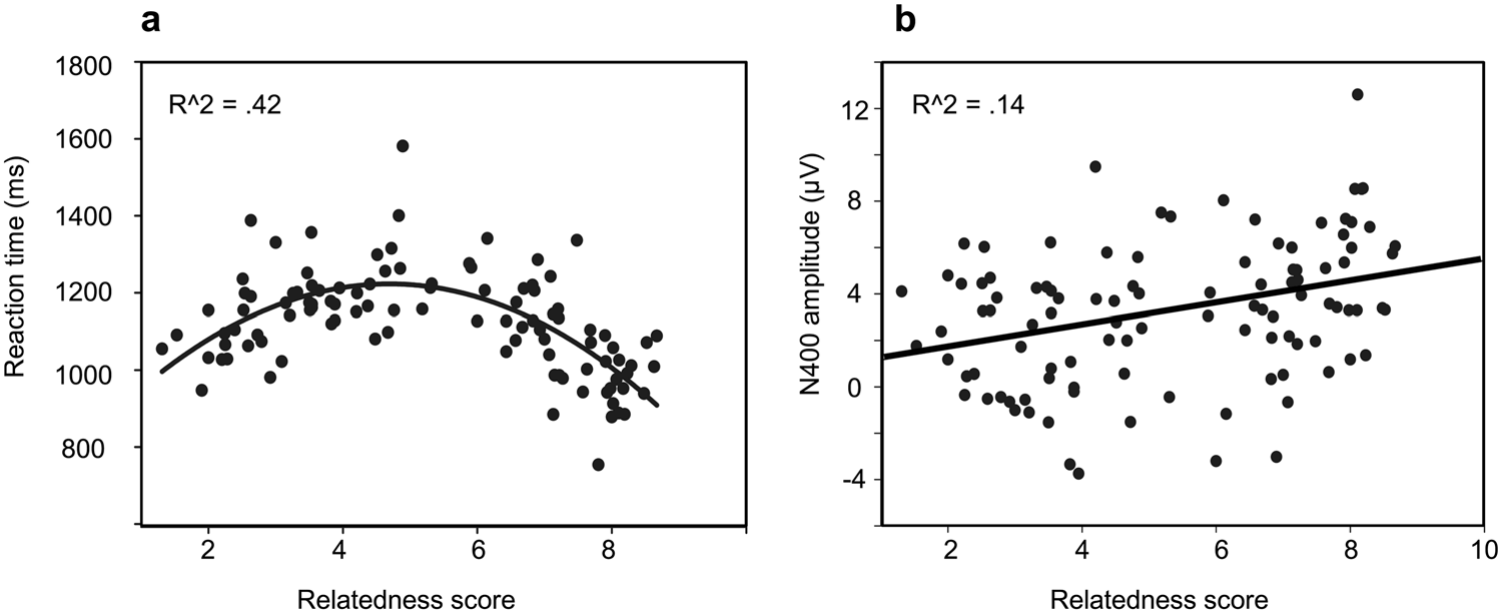
Scatter plots with best-fitting fitted regressions. (**a**) Fitted quadratic regression line for RTs of Exp 2. against the relatedness rating scores of Exp 1. (**b**) Scatter plot and fitted linear regression line for N400 amplitude of Exp 2. against relatedness rating scores of Exp 1.

Finally, to assess whether the pictures elicited the same meaning across different groups of participants we used the scores obtained in the initial rating experiment to predict item-by-item behavioral and N400 responses in the semantic priming experiment. For ‘related’ picture-word pairs, rating scores were negatively correlated with RTs (R = −0.68, *p* < 0.01): the greater the perceived relatedness for a picture-word pair, the faster the response time. Conversely, for ‘unrelated’ picture-word pairs, rating scores were positively correlated with RTs (R = 0.48, *p* < 0.01): the smaller the perceived relatedness for a picture-word pair, the faster the response time. Thus, reaction times were fastest for scores at the extremes of the semantic rating scale and slowest for the items rated in the middle of the scale, likely reflecting degrees of certainty and uncertainty as to the relatedness of given picture-word pairs. Consistent with this interpretation, a quadratic regression of item-based RTs by rating score per condition provided the best fit for the data (R^2^ = 0.42, F_(2,101)_ = 36.6, *p* < 0.001; Fig. 4a). In addition, a regression of item-level N400 amplitude by item-level relatedness score showed a linear relationship, such that an increase in relatedness score predicted a decrease in N400 amplitude (R^2^ = 0.14, F_(1,102)_ = 16.4, *p* < 0.001; Fig. 4b).

## Discussion

Here we set out to test the consistency of semantic relatedness effects in the under-studied case of abstract images. To assess the generic value of abstract images-concepts links, we collected ratings in two independent groups of participants as well as objective measures of semantic priming derived from the well-established N400 effect. Naïve participants who had had no prior exposure to the abstract images used displayed a behavioural priming effect compatible with the ‘related’ – ‘unrelated’ distinction made on the basis of ratings collected in another, independent group of participants. Critically, both this priming effect, and the associated decrease in N400 mean amplitude, correlated with the degree of relatedness established in the preliminary norming procedure. These results show that untimed and explicit relatedness ratings of pictures and labels not only predict how fast different groups of individuals provide a dichotomous rating of abstract picture-word pairs, they also predict the amplitude of brain responses that index meaning processing. To our knowledge, these findings provide the first tangible evidence that abstract images relate consistently to abstract concepts conveyed by words and that they can activate similar such representations across different viewers.

It is an open question whether our findings generalize to any type of abstract image (given that style and origin were essentially random). ERPs do not allow measurement of brain responses elicited by an elaborate description of a painting. Thus, we were limited in the choice of single words that would effectively capture the meaning of an abstract image. This being said, our results can be likened to observations made in the case of music perception where music extracts have been shown to elicit similar semantic representations across listeners^28,29^. We tentatively conclude that concepts activated in long-term memory when one perceives abstract pictures are not purely idiosyncratic to the observer and, on the contrary, have generic relevance. This however, should not imply that individual differences in the appraisal of abstract images are negligible or even modest, since personal experience and aesthetic judgment likely interact in complex ways during image processing^1,30^. In sum, our findings make a compelling case for the idea that abstract images relate to abstract words similarly to the way in which concrete pictures relate to concrete words. This finding may account for the fact that abstract images and textures are widely used in advertising, communication media, and virtual environments, conveying much more it seems than mere aesthetic effects devoid of meaning.

## Methods

### Participants

Online questionnaires were made available to Psychology students at Stirling University, UK for course credits as part of their educational program. The same participant pool was used for the ERP experiment with no participant partaking in more than one experiment. A total of 26 students completed the first questionnaire and 48 the second one. Twenty native English students with no known reading or language impairment participated in the ERP experiment. One dataset was excluded from analysis due to a programming error and one other dataset was excluded due to a self-reported history of brain injury. The remaining 18 participants, 12 female, mean age 20.3; range 17–36; 4 left-handed were included in the analyses. All three experiments (2 rating procedures and the ERP experiment) were performed in accordance with relevant guidelines and regulations and approved by the ethics committee of Stirling University. Informed consent was obtained from all participants before the start of the experiment.

### Materials

We selected 52 images of abstract paintings from online databases and associated these (by experimenter consensus) with abstract verbal labels (See supplementary materials for the stimulus list). These picture–word pairs were presented online (using Qualtrix, www.qualtrics.com) to a group of psychology students (n = 26) along with the question: “How strongly does the word below match the above picture?” (using a 0–10 scale rating) with the option to provide an alternative word. Picture-word pairs with a score of 6 and higher than (52 out of 100) were re-paired to create unrelated picture-word pairs. This set of stimuli was rated by another group of participants (n = 48) resulting in an average score of 7.3, SD = 0.9, range 4.8–8.6 for the related items and an average score of 3.4, SD = 1.0, range 1.3–5.2 for the unrelated items. These ratings were subsequently used for the regression of the item RTs and N400 amplitude in the main experiment.

### Procedure

Internet links of questionnaires were distributed to 1^st^- and 2^nd^-year Psychology undergraduate students at Stirling University. They could fill in the questionnaire at their own pace and received a study token for participation. The data of each questionnaire were collected over three days.

The ERP experiment took place in a sound-attenuated testing booth. Each trial started with presentation of an abstract image, presented full screen on a 17′′ LCD monitor for 5 s. Participants were told that they could freely scan the whole picture, but that they should focus on a centrally placed fixation cross presented after disappearance of the image. The fixation cross was replaced by the target word after 300 ms which in turn disappeared after 1 s while participants had to press a response box button (Psychology Software Tools) to indicate whether or not the picture and the word were related. Response side was counterbalanced across participants and trials were presented in random order. There were short breaks between each of the three 5-minute blocks.

### Data collection

EEG activity was recorded between 0.1 and 200 Hz at a rate of 1 KHz from 64 Ag/Cl electrodes placed according to the extended 10–20 convention with the ground placed between FPz and Fz and the reference between CZ and CPZ. The impedance was kept below 5 KΩ and data was offline re-referenced to the average of left and right mastoid electrodes and filtered using a low-pass, zero-phase shift 30 Hz 48 dB/Oct digital filter. Eye blinks were mathematically corrected using Scan 4.4 (Compumedics, USA) and visually inspected before epochs of −100 to 900 ms in reference to word onset were extracted. Epochs were baseline corrected in reference to pre-stimulus activity and artefacts were removed using a +/−75 µV threshold criterion applied to all electrodes except the vertical electrooculogram.

### ERP analysis

The average number of correctly responded and artefact-free trials included in the analyses was 38 (SD = 5.9; min. 30) in the related and 36 (SD = 7.7; min.20) in the unrelated condition. The difference in number of trials was not significant (p > 0.3). N400 peak detection was conducted predictively at the electrode of maximal sensitivity, CZ between 350–400 ms post stimulus onset. Mean N400 amplitude was calculated over this interval from 15 Fronto-Central-Central-Parietal electrodes classically associated with the N400 (FC1, FC2, FC3, FC4, FCz, C1, C2, C3, C4 Cz, CP1, CP2, CP3, CP4, CPz;)^11^. The same interval and electrodes were used for the item analysis. Individual mean RTs, N400 peak amplitude were subjected to ANOVAs with relatedness (matching vs. unrelated) as within-subjects variable. For the item regression analyses the relatedness ratings obtained from the online questionnaire were used to predict item RT and N400 amplitude of the main experiment.

### Time-frequency analysis

Re-referenced continuous data was filtered low-pass at 100 Hz (24 dB/Oct cutoff), ocular artefacts were rejected using the same procedure as above, epoched into −1 s to 2 s intervals relative to stimulus onset, artefact were rejected as above, before each individual’s average per condition was subtracted from each epoch to create individual trials from which the phase locked ERP data is removed to obtain induced band power^13^. Event-related spectral perturbations for each condition at electrode FCz^14^ were calculated using Morlet wavelets as implemented in EEGLAB^31^. Epochs were split into 200 sliding time windows of 517 ms with 12 ms gaps. Window length was doubled using zero-padding and 1-cycle wavelets were applied to the lowest frequency (resulting in a frequency resolution of ~1 Hz), linearly increasing to 12 cycles at the highest frequency (50 Hz). Log-transformed spectral power was calculated relative to a baseline of −200 to 0 ms relative to word onset during which time a fixation cross had been presented on screen (Fig. 1).

## Electronic supplementary material

Supplementary Table 1

## References

[1] Lasher MD, Carroll JM, Bever TG (1983). The cognitive basis of aesthetic experience. Leonardo.

[2] Leder H, Belke B, Oeberst A, Augustin D (2004). A model of aesthetic appreciation and aesthetic judgments. British journal of psychology.

[3] Pelowski M, Akiba F (2011). A model of art perception, evaluation and emotion in transformative aesthetic experience. New Ideas in Psychology.

[4] Brown C, Hagoort P (1993). The processing nature of the N400: Evidence from masked priming. Journal of cognitive neuroscience.

[5] Luck SJ, Vogel EK, Shapiro KL (1996). Word meanings can be accessed but not reported during the attentional blink. Nature.

[6] Kutas M, Federmeier KD (2011). Thirty years and counting: finding meaning in the N400 component of the event-related brain potential (ERP). Annual review of psychology.

[7] Kutas M, Hillyard SA (1980). Reading senseless sentences: Brain potentials reflect semantic incongruity. Science.

[8] Kutas M, Federmeier KD (2000). Electrophysiology reveals semantic memory use in language comprehension. Trends in cognitive sciences.

[9] Ford JM (1996). N400 evidence of abnormal responses to speech in Alzheimer’s disease. Electroencephalography and clinical Neurophysiology.

[10] Mathalon DH, Faustman WO, Ford JM (2002). N400 and automatic semantic processing abnormalities in patients with schizophrenia. Archives of General Psychiatry.

[11] Wang J, Conder JA, Blitzer DN, Shinkareva SV (2010). Neural representation of abstract and concrete concepts: A meta‐analysis of neuroimaging studies. Human brain mapping.

[12] Marr, D. *Vision* (San Fransisco, CA: Freeman and Sons, 1981).

[13] Ramachandran, V. S. Visual perception in people and machines. In *AI and the Eye* (ed. Blake, A. & Troscianko, T, Chichester: Wiley,1990).

[14] Rorschach, H. *Psychodiagnostics* (Oxford, England: Grune and Stratton, 1942).

[15] Ramachandran VS, Hirstein W (1999). The science of art: A neurological theory of aesthetic experience. Journal of consciousness Studies.

[16] Paivio, A. *Images in mind: The evolution of a theory* (Harvester Wheatsheaf,1991).

[17] Schwanenflugel PJ (1991). Why are abstract concepts hard to understand. The psychology of word meanings.

[18] Barsalou LW (2008). Grounded cognition. Annu. Rev. Psychol..

[19] Binder JR, Desai RH, Graves WW, Conant LL (2009). Where is the semantic system? A critical review and meta-analysis of 120 functional neuroimaging studies. Cerebral Cortex.

[20] Martin, A. Functional neuroimaging of semantic memory in *Handbook of functional neuroimaging of cognition* (eds Calbeza, R. & Kingstone, A) 153–186 (MIT press, 2001).

[21] Martin A (2007). The representation of object concepts in the brain. Annu. Rev. Psychol..

[22] Wilson-Mendenhall CD, Simmons WK, Martin A, Barsalou LW (2013). Contextual processing of abstract concepts reveals neural representations of nonlinguistic semantic content. Journal of cognitive neuroscience.

[23] Kutas, M. & Federmeier, K. D. Thirty Years and Counting: Finding Meaning in the N400 Component of the Event-Related Brain Potential (ERP). A*nnual Review of Psychology* **62** (2011). 10.1146/annurev.psych.093008.131123 PMC4052444 20809790

[24] Klimesch W, Russegger H, Doppelmayr M, Pachinger T (1998). A method for the calculation of induced band power: implications for the significance of brain oscillations. Evoked Potentials-Electroencephalography and Clinical Neurophysiology.

[25] Hagoort P, Hald L, Bastiaansen M, Petersson KM (2004). Integration of word meaning and world knowledge in language comprehension. Science.

[26] Luu P, Tucker DM (2001). Regulating action: alternating activation of midline frontal and motor cortical networks. Clinical Neurophysiology.

[27] Maurer, U. *et al*. Frontal Midline Theta Reflects Individual Task Performance in a Working Memory Task. *Brain Topography* **28** (2015). 10.1007/s10548-014-0361-y 24687327

[28] Koelsch S (2004). Music, language and meaning: brain signatures of semantic processing. Nature Neuroscience.

[29] Peretz I, Zatorre RJ (2005). Brain organization for music processing. Annual Review of Psychology.

[30] Pelowski M, Markey PS, Forster M, Gerger G, Leder H (2017). Move me, astonish me… delight my eyes and brain: The Vienna integrated model of top-down and bottom-up processes in art perception (VIMAP) and corresponding affective, evaluative, and neurophysiological correlates. Physics of Life Reviews.

[31] Delorme, A. & Makeig, S. EEGLAB: an open source toolbox for analysis of single-trial EEG dynamics including independent component analysis. *Journal of Neuroscience Methods* **134** (2004). 10.1016/j.jneumeth.2003.10.009 15102499

