# Supplementary material for: Abstract images and words can convey the same meaning
Source: Sci Rep. 2018 May 8;8:7190. doi: 10.1038/s41598-018-25441-5 (PMC5940816; doi:10.1038/s41598-018-25441-5)
Supplement: Supplementary file 1 — Supplementary Table 1 [file 41598_2018_25441_MOESM1_ESM.pdf]

Supplementary materials

Abstract images and words can convey the same meaning

Jan Rouke Kuipers[1*], Manon Jones[2], Guillaume Thierry[2]

Affiliations:

1 University of Stirling, Stirling FK9 4LA, United Kingdom.

2 Bangor University, Gwynedd LL57 2AS, United Kingdom, g.thierry@bangor.ac.uk.

*Correspondence to:

Psychology Division, Faculty of Natural Sciences,

Stirling University, FK9 4LA Stirling, United Kingdom

E-mail: jk28@stir.ac.uk

Tel.: 0044 1786 466376

Table 1. The stimuli used with the name and author of the work listed where possible.

Artist & Title	Related word	Unrelated word
László Moholy-Nagy, K VII	Organisation	Balance
Unknown	Balance	Hallucination
Unknown	Loneliness	Fertility
Linda C. Deater, Abstract portfolio dlabc055	Chaos	Motion
Unknown	Culture	Growth
Mikalojus Ciurlionis, Allegro (Sonata of the Stars)	Journey	Chaos
Unknown	Hope	Culture
Freydoon Rassouli, gateway to eternity	Creation	Disease
J. Haas: The Galactic Self, Harmonizing World Energies	Energy	Aftermath
Green abstract wallpaper, http://www.wallpapermania.eu	Organicity	Crisis
Unknown	Disease	Expectation
Sven Geier, the road ahead	Regality	Frustration
Maitreyii, unknown	Fluidity	Power
Michelle Warrick, Cool fractals Image detail not the artist/ 14 jewels on https://thereisnocavalry.files.wordpress.com/2012/08/14jewels.jpg	Flamboyance	Doom
Freydoon Rassouli, the voyager	Peacefulness	Conflict
Unknown	Growth	Heat
Unknown	Warmth	Society
Unknown	Haunt	Passion
http://wallpapersinhq.com/images/big/burst_of_light-136544.jpg	Speed	Organisation
Unknown	Celestial	Logic
Unknown	Heat	Play
Willi Boumeister, Unknown	Logic	Excitement
Jeff Lewis, Abstract idealism 2006	Play	Energy
Kazuya Akimoto, The World is originally Geometric	Contrast	Celestial
Unknown	Passion	Journey
Michael McDyer, Angels Fly in the Night	Dread	Organicity
Chua Li Khor, Inner reflections	Tranquillity	Rage
Unknown	Hallucination	Alien
Unknown	Solitude	Warmth
Unknown	Divinity	Speed
Unknown	Motion	Wonder
Moving towards consciousness, exper3.drecursions.com	Wonder	Loneliness
Erin Rafferty, Flare	Expectation	Establishment
Hydrolyphics http://scarart.deviantart.com/art/Abstract-44997773	Power	Peacefulness
CAMartin, Dreams of Dreams	Fertility	Regality
Valkea, 2006	Crisis	Hope

Unknown	Rage	Tranquillity
Richardcgreen, Where Trees Meet the Water and Wet Sand	Alien	Party
Flame design, Whiplash	Vigour	Tranquility
Malahicha, paysage 2	Aftermath	Creation
Pol Ledent, abstract 9645	Conflict	Fluidity
Mark Chadwick, abstract spin painting 23	Excitement	Serenity
Franz Kilne, 1950 study on canvas	Frustration	Vividness
Guilio Baistrocchi, azucar celia cruz baila	Anarchy	Divinity
Dark Abstract art painting (www.thewallpaper.co)	Doom	Vigour
Jackson Pollock, 1949	Noise	Contrast
Vassily Kadinsky, Composition 10	Party	Solitude
Piet Mondriaan, Composition of blue, white yellow and red	Establishmen t	Anarchy
Kimberley Bruce, A Joshua Peace	Tranquility	Flamboyance
Lee Krasner, 1947	Society	Haunt
Mandell, Zoom 14 Satellite Julia Island	Vividness	Dread
Georgia o'Keeffe, Abstraction blue	Serenity	Noise
